# A Bayesian Framework to Identify Methylcytosines from High-Throughput Bisulfite Sequencing Data

**DOI:** 10.1371/journal.pcbi.1003853

**Published:** 2014-09-25

**Authors:** Qing Xie, Qi Liu, Fengbiao Mao, Wanshi Cai, Honghu Wu, Mingcong You, Zhen Wang, Bingyu Chen, Zhong Sheng Sun, Jinyu Wu

**Affiliations:** 1 Institute of Genomic Medicine, Wenzhou Medical University, Wenzhou, China; 2 Beijing Institutes of Life Science, Chinese Academy of Sciences, Beijing, China; 3 Institute of Molecular Medicine, Department of blood transfusion, Zhejiang Provincial People's Hospital, Hangzhou, Zhejiang, China; Ottawa University, Canada

## Abstract

High-throughput bisulfite sequencing technologies have provided a comprehensive and well-fitted way to investigate DNA methylation at single-base resolution. However, there are substantial bioinformatic challenges to distinguish precisely methylcytosines from unconverted cytosines based on bisulfite sequencing data. The challenges arise, at least in part, from cell heterozygosis caused by multicellular sequencing and the still limited number of statistical methods that are available for methylcytosine calling based on bisulfite sequencing data. Here, we present an algorithm, termed Bycom, a new Bayesian model that can perform methylcytosine calling with high accuracy. Bycom considers cell heterozygosis along with sequencing errors and bisulfite conversion efficiency to improve calling accuracy. Bycom performance was compared with the performance of Lister, the method most widely used to identify methylcytosines from bisulfite sequencing data. The results showed that the performance of Bycom was better than that of Lister for data with high methylation levels. Bycom also showed higher sensitivity and specificity for low methylation level samples (<1%) than Lister. A validation experiment based on reduced representation bisulfite sequencing data suggested that Bycom had a false positive rate of about 4% while maintaining an accuracy of close to 94%. This study demonstrated that Bycom had a low false calling rate at any methylation level and accurate methylcytosine calling at high methylation levels. Bycom will contribute significantly to studies aimed at recalibrating the methylation level of genomic regions based on the presence of methylcytosines.

## Author Summary

High-throughput bisulfite sequencing (BS-seq) has advanced tremendously the study of DNA methylation and the determination of methylcytosines at single-base resolution. In BS-seq data analysis, sequencing errors, incomplete bisulfite conversion, and cell heterozygosis affect the accuracy of methylcytosine detection in quite a major way. Simple filtering methods using predefined thresholds have proved to have extremely low efficiency. The commonly used Lister uses binomial distribution to overcome the impacts of non-conversion rate and sequencing errors, but the impact of the cell heterozygosis is not considered. Here, we present Bycom, a novel algorithm based on the Bayesian inference model. To improve the accuracy of methylcytosine calling, Bycom considers sequencing errors, non-conversion rate, and cell heterozygosis integratively to identify methylcytosines from BS-seq data. We evaluated the performance of Bycom using different kinds of BS-seq data. Our results demonstrated that Bycom identified methylcytosines more accurately than Lister, especially in BS-seq data with extremely low genome-wide methylation levels.

## Introduction

DNA methylation is an important epigenetic modification involved in the regulation of gene expression and plays critical roles in cellular processes [1]–[5]. Abnormalities in DNA methylation contribute to the dysregulation of gene expression and have been reported to be associated with tumorigenesis [6] and imprinting disorders [7]. DNA methylation occurs on the cytosine residues in DNA and the accurate identification of methylated cytosines (methylcytosines) is essential for studying variance in methylation [8]. Advances in high-throughput bisulfite sequencing (BS-seq) [9]–[11] such as whole-genome bisulfite sequencing and reduced representation bisulfite sequencing (RRBS), provide comprehensive and well-fitted ways to identify methylcytosines at single-base resolution. However, the large data sets generated by BS-seq pose data processing challenges for methylcytosine calling.

Typically, the first step of methylation analysis with BS-seq data is to map the bisulfite-converted reads to a reference genome using software such as SOAP and BSMAP [12]–[14]. Methylcytosines can then be identified from the reads aligned to the cytosines on the reference genome. However, besides sequencing errors, methylcytosine calling is affected by incomplete bisulfite conversion, which corresponds to the ratio of unmethylated cytosines that were not converted to thymines by the bisulfite treatment. Additionally, cell heterozygosis caused by multicellular sequencing can also influence the precision of methylcytosine detection because the methylation status of the same cytosine site in different cell is probably inconsistent owing to the coexistence of methylation and demethylation [15]–[18].

As a consequence of the factors mentioned above, the false-positive rate for methylcytosines detected by simple filtering using a predefine threshold that the methylation level should be above zero, has been reported to be extremely high [19]–[21]. In recent years, the methylation ascertainment method applied by Lister et al. [9], which is widely used in the methylation analysis software such as Bismark [22] and Bisulfighter [23], has been employed to determine the methylcytosines from BS-seq data [5], [24] and has been shown to have a much lower false positive rate than the simple filtering method [25], [26]. We termed the method as “Lister” hereafter. Although Lister uses binomial distribution to overcome the impacts of false-positive rate (sum of non-conversion rate and sequencing errors) [24], it does not consider the impact of methylation heterozygosis caused by cell heterozygosis. Besides, as amounts of cytosines, whose methylation level is low, are difficult to be distinguished from unmethylated sites, the methylation status determined by the binomial test is not reliable for samples with extremely low genome-wide methylation levels [5].

Here, we present a novel algorithm, Bycom that can take into account sequencing errors, non-conversion rate, and cell heterozygosis which is initially introduced as the factors to identify precisely the methylcytosines from BS-seq data. Bycom is based on the Bayesian inference model, which is not limited to certain data distribution and has been used successfully for single-nucleotide polymorphism (SNP) calling in next-generation sequencing data [27]. Bayesian inference model has also been successfully applied to methylated DNA immunoprecipitation (MeDIP)-based studies [28]. We evaluated the performance of Bycom on simulated BS-seq data, publicly available BS-Seq data, and RRBS data and demonstrated that Bycom can identify methylcytosines more precisely than Lister. This methodology of Bycom has been packaged and available at https://sourceforge.net/projects/bycom, which is written in PERL and designed for Linux 64 platform.

## Results

**Figure 1:**
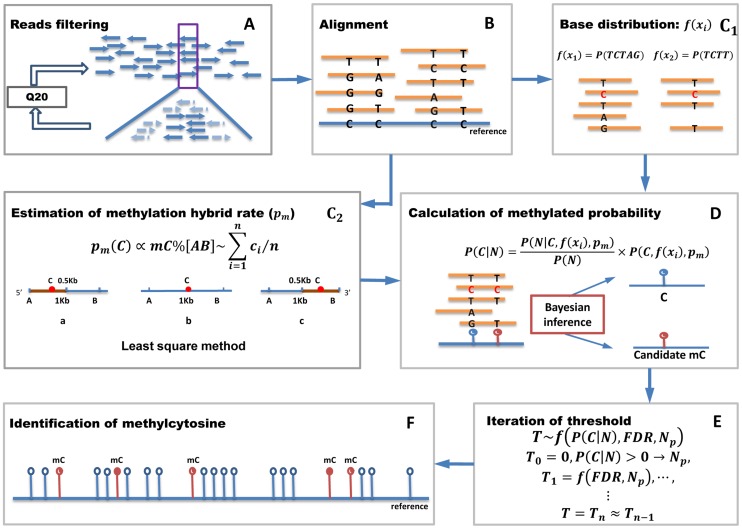
Overview of the Bycom workflow for methylcytosine calling. (**A**) Low quality reads filtered by Q20 value. (**B**) Filtered reads mapped to the reference genome. (**C**) Base distribution 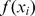 (C_1_) and methylation hybrid rate P_m_ (C_2_) were estimated from the mapping results. P_m_ was evaluated from the methylation level of a 1000-bp region centered on selected cytosine sites. For cytosine sites in the 500-bp head or tail of a chromosome, P_m_ was calculated as the average methylation level of the 1000-bp head or tail of the chromosome. (**D**) Based on the 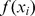 and P_m_, the true positive sites were identified as candidate methylcytosines using a Bayesian inference model from the false positive sites with methylation levels above zero. (**E, F**) Methylcytosines identified using iteration of a set threshold.

The Bycom procedures used to identify methylcytosines from high-throughput BS-seq data are depicted in Figure 1. First, the raw reads generated by BS-seq were filtered using a Q20 cut-off to exclude low quality reads whose average ASCII quality values were less than the char “T” (Figure 1A). Second, the filtered reads were aligned to a reference genome sequence using mapping software BSMAP (Figure 1B). In this step, cytosine sites with quality values less than Q20, which indicated sequencing errors, were further excluded. Third, after filtering, the base distribution in a 1000-bp window was used to calculate the average methylation level in the region covered by the window [24], [28], [29]. Based on the calculated average methylation level, a least square method was used to evaluate the methylation heterozygosity (Figure 1C). Using the base distribution and methylation heterozygosity information, the posterior probability of the nucleotide at the reference cytosine was calculated using a Bayesian inference formula (Figure 1D). The cytosines that have the highest posterior probabilities (methylated probability) were selected as candidate methylcytosines (Figure 1E). Finally, methylcytosines were determined from the candidate methylcytosines using an iterated threshold method (Figure 1F). Meanwhile, Lister utilizes the mapping results of BSMAP to conduct the binary classification of mCs [9].

### Performance of Bycom on simulated data

**Figure 2:**
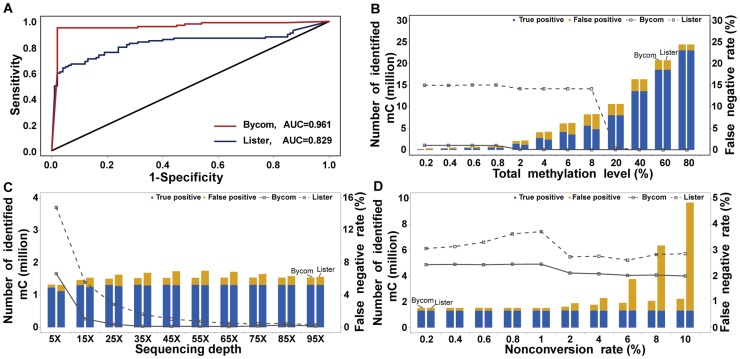
Effects of sequencing depth, non-conversion rate, and methylation level on Bycom performance on simulated data. The performance of Bycom was compared with the performance of Lister. (**A**) Receiver operator characteristic (ROC) curves for Bycom and Lister with all the factor parameters set at the default values. (**B, C, D**) Performance of Bycom and Lister with a range of values for the sequencing depth parameter (B), the non-conversion rate parameter (C), and the methylation level parameter (D). Histograms indicate the number of identified methylcytosines, and lines indicate the false negative rate.

The performance of Bycom was evaluated and compared with Lister based on simulated data that were generated from chromosome 10 of the human reference genome sequence (hg18, UCSC) using the bisulfite sequencing simulator for next-generation sequencing BSSim (http://122.228.158.106/BSSim). Six factors that may affect the identification of methylcytosines were considered in the simulation process, namely, total methylation level of the sequenced sample, sequencing depth, bisulfite conversion rate, high quality value ratio, mismatch number, and SNP frequency. With all parameters set to their default values, we found that Bycom had a higher overall accuracy than Lister as shown by the receiver operator characteristic (ROC) curves (Figure 2A).

Next, we investigated the impact of each single factor on the performance of Bycom. For the total methylation level, we assumed that the number of methylcytosines in the simulated data increased steadily as the total methylation level was increased. When the methylation levels were extremely low (0.2%, 0.4%, 0.6%, and 0.8%), the number of methylcytosines called rose slightly and the false negative rate remained stable at 2% for Bycom and at 15% for Lister. At low methylation levels (2%, 4%, 6%, and 8%), the number of methylcytosines called by the two methods increased and the false negative rate for Bycom decreased slightly to 0.3% compared with 14% for Lister. At the high methylation levels (20%, 40%, 60%, and 80%), the number of true positive sites increased, while the false negative rates for both Bycom and Lister remained very low (Figure 2B).

For the impact of sequencing depth, the total number of methylcytosines called by Bycom increased when the read depth incrementally changed from 5× to 25×. The highest number of methylcytosines called was when the depth was 25×; thereafter, the number of calls remained relatively constant for depths >25× (Figure 2C). In contrast, the total number of methylcytosines called by Lister increased when the read depth went up from 5× to 55× after which the number of calls steadily decreased. The false negative rates for both Bycom and Lister reduced when the higher read depths were introduced and, in all cases, the false positive rates for Lister were higher than the false positive rates for Bycom.

The bisulfite conversion rate can affect the methylation level of the cytosines in a genome sequence; therefore, we evaluated the performance of Bycom using different values for this parameter. The number of methylcytosines called by Bycom and Lister remained constant when the non-conversion rate was <1%. When the non-conversion rate was >1%, the total number of methylcytosines called by Bycom increased slightly and the false negative decrease steadily; conversely, the total number of methylcytosines called by Lister increased sharply (Figure 2D). The other three factors, high quality value ratio (Q20 cut-off), mismatch number, and SNP frequency made only small contributions to either the number of methylcytosines called or the false positive rate, both of which remained nearly constant. Here, for the method Lister, the influence of sequencing errors calculated by quality value were reduced as the quality cut-off went up, while for Bycom, the influence was still kept at a relatively low level (Figure S1B & S1D). Overall, compared with the performance of Lister, Bycom generated more true positive sites and fewer false positive sites (Figure S1).

### Performance of Bycom on publicly available BS-seq data with known methylcytosines

**Figure 3:**
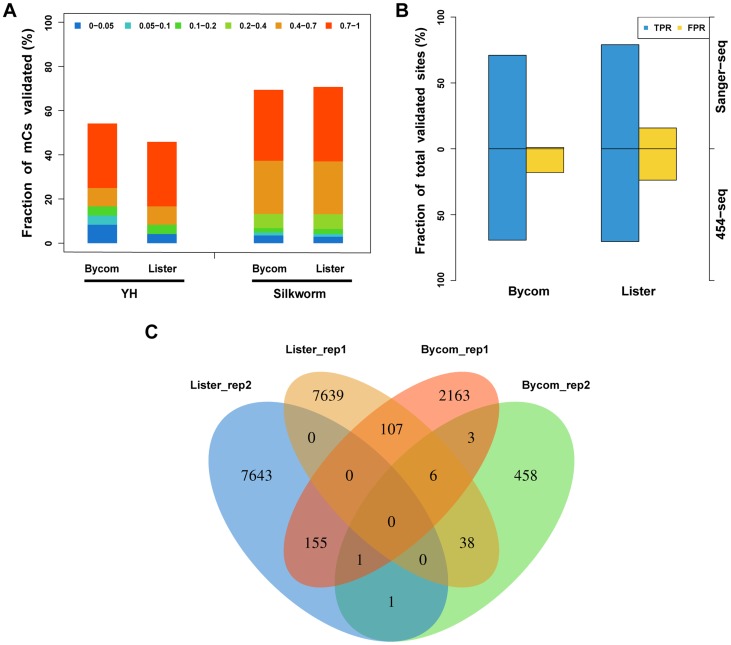
Performance of Bycom on YH data, silkworm data, and *Aspergillus flavus* data. (**A**) Distribution of validated methylcytosines called by Bycom compared with those called by Lister using YH and silkworm data; Colors stand for the fraction of validated methylcytosines with different methylation levels. (**B**) Distribution of the validated sites called by Bycom compared with those called by Lister using silkworm data that were validated by 454 sequencing and/or Sanger sequencing. TPR, true positive rate; FPR, false positive rate. (**C**) Called methylcytosine sites that overlapped between Bycom and Lister using *Aspergillus flavus* data that contained two biological replications.

To further assess the ability of Bycom to identify methylcytosines, whole-genome BS-seq data of YH [24], silkworm [19] and the ascomycetes *Aspergillus flavus* [5] were used as described in [24]. The YH BS-seq data, generated from the peripheral blood mononuclear cells of an Asian individual, had a high methylation level of 68.4% at CpG sites and <0.2% at non-CpGs sites with an overall coverage of 12.3-fold per strand. 32 methylated CpGs and 18 non-methylated CGs were selected randomly and validated by bisulfite Sanger sequencing. Because the depth of 11 validated cytosines was lower than the threshold (lowest depth was 4-fold) [24], only 24 methylated CpGs and 15 non-methylated CGs gave eligible validated results. The methylcytosine calling results showed that the accuracy for Bycom was 71.79% compared with the 66.67% accuracy for Lister, and both methods made no false calls (Figure 3A).

We also evaluated the performance of Bycom on a species that had a low methylation level. The BS-seq data of silkworm had overall methylation levels of 0.67% at CG, 0.21% at CHG, and 0.24% at CHH sites (where H is A, C, or T) with an average depth of 7.4-fold per strand. In the BS-seq silkworm data, 598 methylcytosines and 311 cytosines were validated (≥4×) among which 24 methylated and 101 non-methylated sites were verified by bisulfite Sanger sequencing while the others were confirmed using bisulfite-PCR followed by 454 sequencing. The distribution of the validated sites called by Bycom and Lister is shown in Figure 3A. For bisulfite Sanger sequencing, Bycom had 93.6% accuracy with a false positive rate of 0.99%, while Lister exhibited 83.2% accuracy with a false positive rate of 15.84%. For 454 sequencing, the Bycom and Lister accuracies were the same at 82.53% with false positive rates of 10.48% for Bycom and 13.81% for Lister (Figure 3B).

Finally, we evaluated the performance of Bycom on the BS-seq data of *Aspergillus flavus*, which had been reported to have a negligible level of methylation with 4-fold overall depth per strand. All the methylcytosines called by Lister on this data were regarded as false positives (≥4×). An overlapping strategy was applied between the cytosines detected by Bycom and by Lister. The results indicated that the false positive rate for Lister was at least three times the false positive rate for Bycom (Figure 3C). Further, sites that were selected randomly from the methylcytosines called by Lister and verified by Sanger sequencing as non-methylated cytosines were not called by Bycom.

Meanwhile, we splited the validated sites of YH, silkworm and *Aspergillus flavus* into CpG and CpH (CpA, CpC, and CpT dinucleotides) context. The strategy was also performed on the validated results of Bycom and Lister in these samples. We found that true positive sites (mC) were all in CpG context and the sites in CpH context were all false positive. The results also indicated that Bycom was more precise on the detection of mCpH than Lister (Table S1).

### Performance of Bycom on RRBS data from human ovarian epithelial T29 cells

We evaluated the performance of Bycom on RRBS data from T29 cells (GSE55568). A total of 2,110,089 methylcytosines were identified in the genome, making up nearly 0.18% of the total cytosines; 98.58% of the identified methylcytosines were in CG sites with a mean sequencing depth of 15.5-fold per strand (Table S2).

To verify the precision of Bycom, seven batches of sequences from the T29 RRBS data were selected randomly for validation by bisulfite-PCR followed by sequencing on the Illumina MiSeq platform. As a result, 68 methylcytosines and 158 cytosines were validated. Bycom identified a high percentage (75%) of the CpG methylcytosines and a low percentage (5.56%) of the CpH methylcytosines. Lister identified a relatively low percentage (71.88%) of mCpG and the equal proportion of mCpH as Bycom. Besides, a similar low proportion of the validation sites were identified as cytosines by both Bycom and Lister (Table S3). Bycom and Lister had an accuracy of 93.81% and 93.36%, respectively, with the same false positive rate of 4.12%. Notably, methylcytosines in the CpH context, which were nearly complete absence in the T29 cells [20], were regarded as background noise (Table S3).

### Comparison of Bycom to Bismark and Bisulfighter

**Figure 4:**
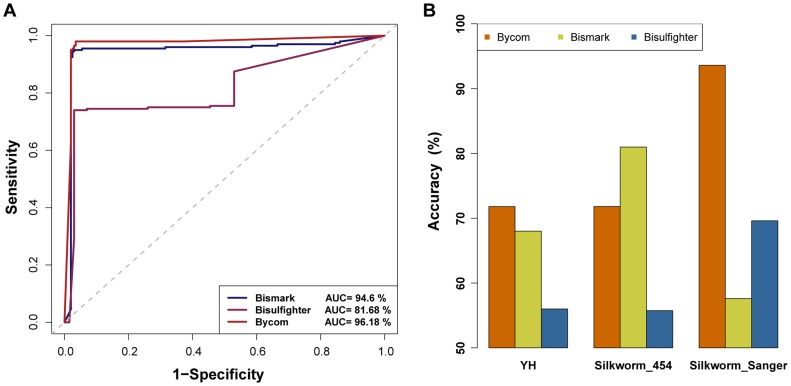
Comparison of the software based on Bycom to Bismark and Bisulfighter on simulation data and public data. (**A**) ROC curves for Bycom, Bismark and Bisulfighter with all the factor parameters set at the default values. (**B**) Accuracy of Bycom, Bismark and Bisulfighter on the validated results of YH Sanger-sequencing data, silkworm Sanger-sequencing data and silkworm 454-sequencing data.

We compared the accuracy of mC calling between Bycom and methylation analysis tools, including Bismark and Bisulfighter, which employed *Lister* as the algorithm to detect methylcytosines. With all parameters set to default in simulator BSSim, we found that the software based on Bycom run fastest (Table S4). Besides, Bycom had a highest overall accuracy than others as shown by the ROC curves (Figure 4A). Then, we compared the software based on Bycom with Bismark and Bisulfighter using the previous public data. Firstly, Bycom had a highest accuracy than other two in YH Sanger-sequencing data without false positive results. Secondly, in silkworm data, for 454-sequencing validation, the accuracy of Bycom was lower than that of Bismark and higher than that of Bisulfighter. For Sanger-sequencing validation, Bycom kept highest precision on the detection of methylcytosines than others (Figure 4B). Finally, the validation sites of *Aspergillus flavus* Sanger sequencing data all proven to be false positive were not called by Bycom and Bismark, and 16.67% of them were selected by Bisulfighter as methylcytosines.

## Discussion

Detecting methylcytosines accurately from high-throughput sequencing data is an essential step to calculate the methylation level of genomes at single-base resolution. In this study, we presented a novel computational strategy, Bycom, for identifying precisely methylcytosines from BS-seq data using the Bayes inference model. Bycom not only considers the impacts of sequencing errors and non-conversion rate, both of which are treated as the false-positive rate in the Lister method, but also introduces cell heterozygosis to identify methylcytosines in an unbiased manner.

The results on the simulated data showed that the non-conversion rate and total methylation level significantly affected the accuracy of the Bycom and Lister methods, while sequencing errors had limited influence after the relatively stringent quality filtration of the data. Additionally, the accuracy of methylcytosine calling was better with Bycom than with Lister. The results on the public data indicated that the false positive rate of Lister was higher than the false positive rate of Bycom in the genome-wide samples with low methylation levels. Although The validation sites of public data in CpH context were all detected as false positive sites, Bycom had a better performance on the classification of mCpH than Lister. Furthermore, the validation results on the T29 RRBS data showed that Bycom detected the vast majority of methylcytosines and maintained the false calling rate at a low level. The performance of Bycom on the public data with different genome-wide methylation levels revealed that Bycom had no bias on data with high or low methylation levels. Moreover, to further verify the accuracy of our method, we compared the accuracy of Bycom with the methylation analysis tools, Bismark and Bisulfighter, which could also be used to call methylcytosines. The results of simulated data and public data both showed that Bycom behaved better than Bismark and Bisulfighter.

However, we noticed that variances of the methylation level on single cytosines between the BS-seq data and the Sanger/454 sequencing data likely led to false/missing calls by both Bycom and Lister. Although the methylcytosines were still detected by Bycom, this finding suggested that the accuracy might improve further if the methylation level of the base was recalibrated depending on mass validated sequencing data. In future work, we aim to refine the model with well-recalibrated sequencing data. Besides, other functions, such as differentially methylated regions (DMRs) detection will be added in future, which will be great facilitated by the precise methylated cytosines calling. In conclusion, the results presented here demonstrate that Bycom is an effective statistical framework that is capable of identifying methylcytosines and will be very useful for genome-wide investigations of DNA methylation.

## Materials and Methods

### Data sets

NCBI build 36.1 (hg18) was downloaded from the UCSC database (http://genome.ucsc.edu/) and used as the human reference genome. The whole-genome DNA methylation data from an anonymous male Asian Han Chinese (YH) were obtained from the NCBI Gene Expression Omnibus (GEO) (http://www.ncbi.nlm.nih.gov/geo; GSE17972). The silkworm sequence data were downloaded from the GEO database (GSE18315) and the Sequence Read Archive (SRA; SRP001159). The *Aspergillus flavus* reference genome sequence was downloaded from the *Aspergillus flavus* Genome Sequencing Project web site (http://www.aspergillusflavus.org/genomics/) and the DNA methylation data were obtained from GEO (GSE32177). The raw Illumina T29 RRBS data are available from GEO under accession number GSE55568.

### Simulated data

Simulated bisulfite short reads with different levels of six factors (total methylation level, sequencing depth, bisulfite conversion rate, sequencing quality of base, number of misaligned reads, and mutation rate) were simulated from chromosome 10 of hg18 using BSSim (http://122.228.158.106/BSSim/). The default settings of the six factors in BSSim were used for the simulation as: sequencing depth 30×; maximum mismatches 2; bisulfite conversion rate 0.998; high quality value ratio 95%; and SNP frequency for homozygote/heterozygote 0.0005/0.001. Besides, different methylation levels of 86.53%, 2.03%, and 2.27% were set for the different types of cytosines CG, CHG, and CHH, respectively, where H is A, C, or T.

Reads were generated from random locations on chromosome 10 with different settings of the six factors; one factor was changed while the other parameters remained at the default values. Different levels were set as: sequencing depth 5×, 15×, 25×, …, 95×; mismatch number 1, 2, 3, …, 10; bisulfite non-conversion rate 0.01, 0.02, 0.03, …, 0.1; and whole-genome methylation level 0.002, 0.004, 0.006, 0.008 (extremely low), 0.02, 0.04, 0.06, 0.08 (low), and 0.2, 0.4, 0.6, 0.8 (high). For SNP frequency, homozygotes were set as 0.0005, 0.001, 0.0015, …, 0.005 and heterozygotes were set at double the homozygote frequency. High quality values indicated by ASCII quality values above the char “h” of 10%, 20%, 30%, …, 90%, 99% were assigned to the whole reads.

The methylation status and SNP genotypes of each cytosine were then retrieved from the BSSim simulated files.

### Evaluation of read mapping and base distribution

Low quality reads (Q20) were eliminated for the raw data and the clean reads were aligned against the reference genome allowing a maximum of two mismatches. Uniquely mapped reads were retained for the following analysis. For a selected genomic location, the cytosines in that region were set as 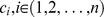, where n is the total number of cytosines in the region. Then, the observed base of a mapped read j at cytosine site c_i_ in the genomic sequence was assumed as 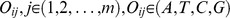, where m is the total number of reads that cover site 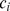. The quality value of the observed base 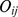 was set to 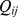.

### Basic statistical model

In the Bayesian inference model, the posterior probability of expecting base 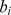 at site 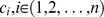 on the reference genome was expressed as:
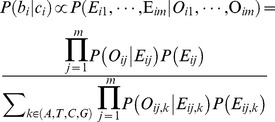
where m is the total number of reads covering site 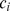. Here, 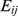 was considered as the actual base according to the observed base 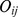 of the read when *b_i_* was estimated. The independent base was expressed as 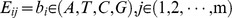. When the sequencing error ratio is set to 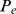, the likelihood 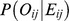 of the site 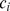 was calculated as:
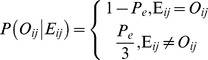



Beside the sequencing error, other factors, including bisulfite conversion rate 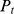 and methylated heterozygosity 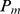, also influence the composition of the bases derived from the reads. After bisulfite treatment, heterozygous methylcytosines 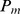, heterozygous cytosines 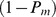, wrong-sequenced cytosines 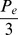, correct-sequenced cytosines 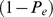, converted cytosines 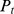, and unconverted cytosines 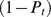 may appear simultaneously at identical cytosine sites in the sequences from distinct cells. Therefore, the likelihood 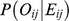 was computed as:
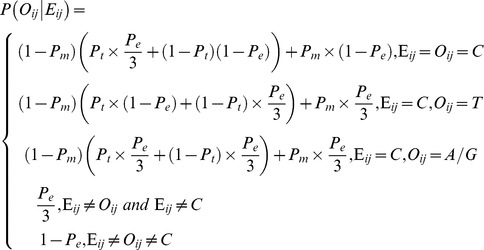
The prior probability 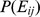 of each base *b_i_* at site 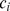 on the genome was set as:
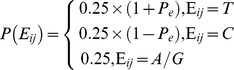



In this way, the posterior probability derived from the Bayesian inference formula was obtained.

### Parameter estimation

The key to the model is the accurate estimation of three parameters, 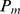, 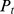 and 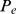, which can change the of base contribution component from the reads covering the cytosine site. To estimate the methylated heterozygosity 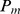, a linear fitting equation was built using the methylation level of a 1000-bp genome region of single chromosome in which the methylation status has been reported to be highly correlated [24], [28]. The 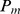 of the midpoint in a 1000-bp region was estimated as:
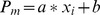
where 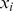 is the methylation level (methylated reads/total reads) of site 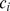. The values of a and b were obtained by a least square procedure as:
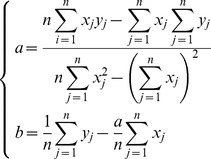
where 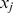 is the methylation level of site 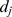 in a 1000-bp region centered at sites 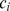 and 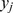 is the average methylation level of the 1000-bp region centered at sites 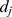. The region centered at 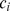 contained n cytosine sites. The 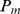 of cytosine sites in the 500-bp head or tail of the chromosome were estimated using the average methylation level of the cytosine sites in the 1000-bp head or tail chromosome genome regions as:
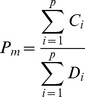
Where p is the total number of the cytosine sites located in the 1000-bp head or tail of the chromosome. 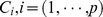 and 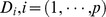 are the number of methylated reads and total reads at site 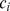, respectively. The sequencing error ratio 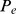, which is indicated by the quality value 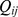 of the observed base 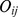, was calculated as 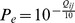 [30]. The bisulfite conversion rate 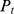 was determined initially by un-methylated lambda DNA spike-ins [25] or by calculating the C to T conversion rate for all cytosines in the CpH context [19], [24].

### Methylcytosine calling

After bisulfite treatment, the posterior probability of confirming the cytosine site as base C is actually the probability of cytosine methylation. The threshold T was set to ensure methylcytosine calling was precise, and was computed by a numerical iteration algorithm as:
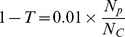
(*)where 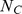 and 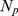 represent the number of cytosine and methylcytosine sites, respectively, on a chromosome. The false discovery rate (FDR) was set as 0.01. In the iteration, 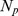 was calculated as the number of cytosine sites with a posterior probability above the T threshold. Set 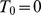 for the first iteration, and the posterior probability above the value 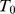 of the cytosine would be the methylated cytosine. Then we can calculate 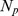, substitute it into (*) and obtain 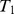. For the second iteration, 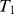 replaces 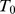, and acquire 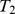; 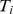 will tend to the invariant threshold as T after several iterations.

### Validation

Total genomic DNA was extracted from the T29 cell line and converted by sodium bisulfite following an established protocol [31]. Seven batches of regions were selected randomly and amplified with nested RT-PCR primers (Table S5). The amplified products were sequenced on the Illumina MiSeq platform (Illumina Inc, San Diego, CA). The validation results are summarized in Table S3.

## Supporting Information

Figure S1 **Effects of high quality value ratio (Q20), high quality value ratio (Q0), mismatch number, and SNP frequency on Bycom performance on simulated data.** Performance of Bycom and Lister with a range of values for the mismatch number (**A**), the high quality value ratio using Q20 cut-off (**B**), the SNP frequency (**C**), and the high quality value ratio using Q0 cut-off (**D**). Histograms indicate the number of identified methylcytosines, and lines indicate the false negative rate.
(TIF)

Figure S2 **Statistic details about the methylcytosines calling on YH data.** (**A**) Methylation level distribution of mCpGs called by Bycom and Lister. (**B**) Distribution of the mCpGs called by Bycom and Lister on the fuctional elements of the genome. (**C**) The percent of mCpGs, detected by Bycom and Lister, in different methylation level on the fuctional elements of the genome.
(TIF)

Table S1 **The validation results of Bycom and Lister on public data.**
(XLSX)

Table S2 **Summary of T29 RRBS data production.**
(XLSX)

Table S3 **Summary of data validation.**
(XLSX)

Table S4 **Computational cost of Bycom, Bismark and Bisulfighter on simulated data.** Computation time was evaluated based on 10G simulated reads.
(XLSX)

Table S5 **Primers used for bisulfite-PCR.**
(XLSX)

Text S1 **Commands of the public software used in this manuscript.**
(DOCX)

## References

[1] Feinberg AP (2007) Phenotypic plasticity and the epigenetics of human disease. Nature 447: 433–440. 17522677 10.1038/nature05919

[2] Robertson KD (2005) DNA methylation and human disease. Nat Rev Genet 6: 597–610. 16136652 10.1038/nrg1655

[3] Kaminsky Z, Tochigi M, Jia P, Pal M, Mill J, et al (2012) A multi-tissue analysis identifies HLA complex group 9 gene methylation differences in bipolar disorder. Mol Psychiatry 17: 728–740. 21647149 10.1038/mp.2011.64

[4] Iwai M, Kiyoi H, Ozeki K, Kinoshita T, Emi N, et al (2005) Expression and methylation status of the FHIT gene in acute myeloid leukemia and myelodysplastic syndrome. Leukemia 19: 1367–1375. 15902282 10.1038/sj.leu.2403805

[5] Liu SY, Lin JQ, Wu HL, Wang CC, Huang SJ, et al (2012) Bisulfite sequencing reveals that Aspergillus flavus holds a hollow in DNA methylation. PLoS ONE 7: e30349. 22276181 10.1371/journal.pone.0030349 PMC3262820

[6] Heard E, Clerc P, Avner P (1997) X-chromosome inactivation in mammals. Annu Rev Genet 31: 571–610. 9442908 10.1146/annurev.genet.31.1.571

[7] Li E, Beard C, Jaenisch R (1993) Role for DNA methylation in genomic imprinting. Nature 366: 362–365. 8247133 10.1038/366362a0

[8] Smallwood SA, Tomizawa S, Krueger F, Ruf N, Carli N, et al (2011) Dynamic CpG island methylation landscape in oocytes and preimplantation embryos. Nat Genet 43: 811–814. 21706000 10.1038/ng.864 PMC3146050

[9] Lister R, Pelizzola M, Dowen RH, Hawkins RD, Hon G, et al (2009) Human DNA methylomes at base resolution show widespread epigenomic differences. Nature 462: 315–322. 19829295 10.1038/nature08514 PMC2857523

[10] Cokus SJ, Feng S, Zhang X, Chen Z, Merriman B, et al (2008) Shotgun bisulphite sequencing of the Arabidopsis genome reveals DNA methylation patterning. Nature 452: 215–219. 18278030 10.1038/nature06745 PMC2377394

[11] Zhong S, Fei Z, Chen YR, Zheng Y, Huang M, et al (2013) Single-base resolution methylomes of tomato fruit development reveal epigenome modifications associated with ripening. Nat Biotechnol 31: 154–159. 23354102 10.1038/nbt.2462

[12] Krueger F, Kreck B, Franke A, Andrews SR (2012) DNA methylome analysis using short bisulfite sequencing data. Nat Methods 9: 145–151. 22290186 10.1038/nmeth.1828

[13] Xi Y, Li W (2009) BSMAP: whole genome bisulfite sequence MAPping program. BMC Bioinformatics 10: 232. 19635165 10.1186/1471-2105-10-232 PMC2724425

[14] Li R, Li Y, Kristiansen K, Wang J (2008) SOAP: short oligonucleotide alignment program. Bioinformatics 24: 713–714. 18227114 10.1093/bioinformatics/btn025

[15] Wu SC, Zhang Y (2010) Active DNA demethylation: many roads lead to Rome. Nat Rev Mol Cell Biol 11: 607–620. 20683471 10.1038/nrm2950 PMC3711520

[16] Okada Y, Yamagata K, Hong K, Wakayama T, Zhang Y (2010) A role for the elongator complex in zygotic paternal genome demethylation. Nature 463: 554–558. 20054296 10.1038/nature08732 PMC2834414

[17] Ooi SK, Qiu C, Bernstein E, Li K, Jia D, et al (2007) DNMT3L connects unmethylated lysine 4 of histone H3 to de novo methylation of DNA. Nature 448: 714–717. 17687327 10.1038/nature05987 PMC2650820

[18] Jia D, Jurkowska RZ, Zhang X, Jeltsch A, Cheng X (2007) Structure of Dnmt3a bound to Dnmt3L suggests a model for de novo DNA methylation. Nature 449: 248–251. 17713477 10.1038/nature06146 PMC2712830

[19] Xiang H, Zhu J, Chen Q, Dai F, Li X, et al (2010) Single base-resolution methylome of the silkworm reveals a sparse epigenomic map. Nat Biotechnol 28: 516–520. 20436463 10.1038/nbt.1626

[20] Ziller MJ, Muller F, Liao J, Zhang Y, Gu H, et al (2011) Genomic distribution and inter-sample variation of non-CpG methylation across human cell types. PLoS Genet 7: e1002389. 22174693 10.1371/journal.pgen.1002389 PMC3234221

[21] Stevens M, Cheng JB, Li D, Xie M, Hong C, et al (2013) Estimating absolute methylation levels at single-CpG resolution from methylation enrichment and restriction enzyme sequencing methods. Genome Res 23: 1541–1553. 23804401 10.1101/gr.152231.112 PMC3759729

[22] Krueger F, Andrews SR (2011) Bismark: a flexible aligner and methylation caller for Bisulfite-Seq applications. Bioinformatics 27: 1571–1572. 21493656 10.1093/bioinformatics/btr167 PMC3102221

[23] Saito Y, Tsuji J, Mituyama T (2014) Bisulfighter: accurate detection of methylated cytosines and differentially methylated regions. Nucleic Acids Res 42: e45. 24423865 10.1093/nar/gkt1373 PMC3973284

[24] Li Y, Zhu J, Tian G, Li N, Li Q, et al (2010) The DNA methylome of human peripheral blood mononuclear cells. PLoS Biol 8: e1000533. 21085693 10.1371/journal.pbio.1000533 PMC2976721

[25] Gao F, Liu X, Wu XP, Wang XL, Gong D, et al (2012) Differential DNA methylation in discrete developmental stages of the parasitic nematode Trichinella spiralis. Genome Biol 13: R100. 23075480 10.1186/gb-2012-13-10-r100 PMC4053732

[26] Lyko F, Foret S, Kucharski R, Wolf S, Falckenhayn C, et al (2010) The honey bee epigenomes: differential methylation of brain DNA in queens and workers. PLoS Biol 8: e1000506. 21072239 10.1371/journal.pbio.1000506 PMC2970541

[27] Li R, Li Y, Fang X, Yang H, Wang J, et al (2009) SNP detection for massively parallel whole-genome resequencing. Genome Res 19: 1124–1132. 19420381 10.1101/gr.088013.108 PMC2694485

[28] Down TA, Rakyan VK, Turner DJ, Flicek P, Li H, et al (2008) A Bayesian deconvolution strategy for immunoprecipitation-based DNA methylome analysis. Nat Biotechnol 26: 779–785. 18612301 10.1038/nbt1414 PMC2644410

[29] Eckhardt F, Lewin J, Cortese R, Rakyan VK, Attwood J, et al (2006) DNA methylation profiling of human chromosomes 6, 20 and 22. Nat Genet 38: 1378–1385. 17072317 10.1038/ng1909 PMC3082778

[30] Fujimoto A, Nakagawa H, Hosono N, Nakano K, Abe T, et al (2010) Whole-genome sequencing and comprehensive variant analysis of a Japanese individual using massively parallel sequencing. Nat Genet 42: 931–936. 20972442 10.1038/ng.691

[31] Grunau C, Clark SJ, Rosenthal A (2001) Bisulfite genomic sequencing: systematic investigation of critical experimental parameters. Nucleic Acids Res 29: E65–65. 11433041 10.1093/nar/29.13.e65 PMC55789

