# Supplementary figures and images for: A Bayesian Framework to Identify Methylcytosines from High-Throughput Bisulfite Sequencing Data
Source: PLoS Comput Biol. 2014 Sep 25;10(9):e1003853. doi: 10.1371/journal.pcbi.1003853 (PMC4177668; doi:10.1371/journal.pcbi.1003853)

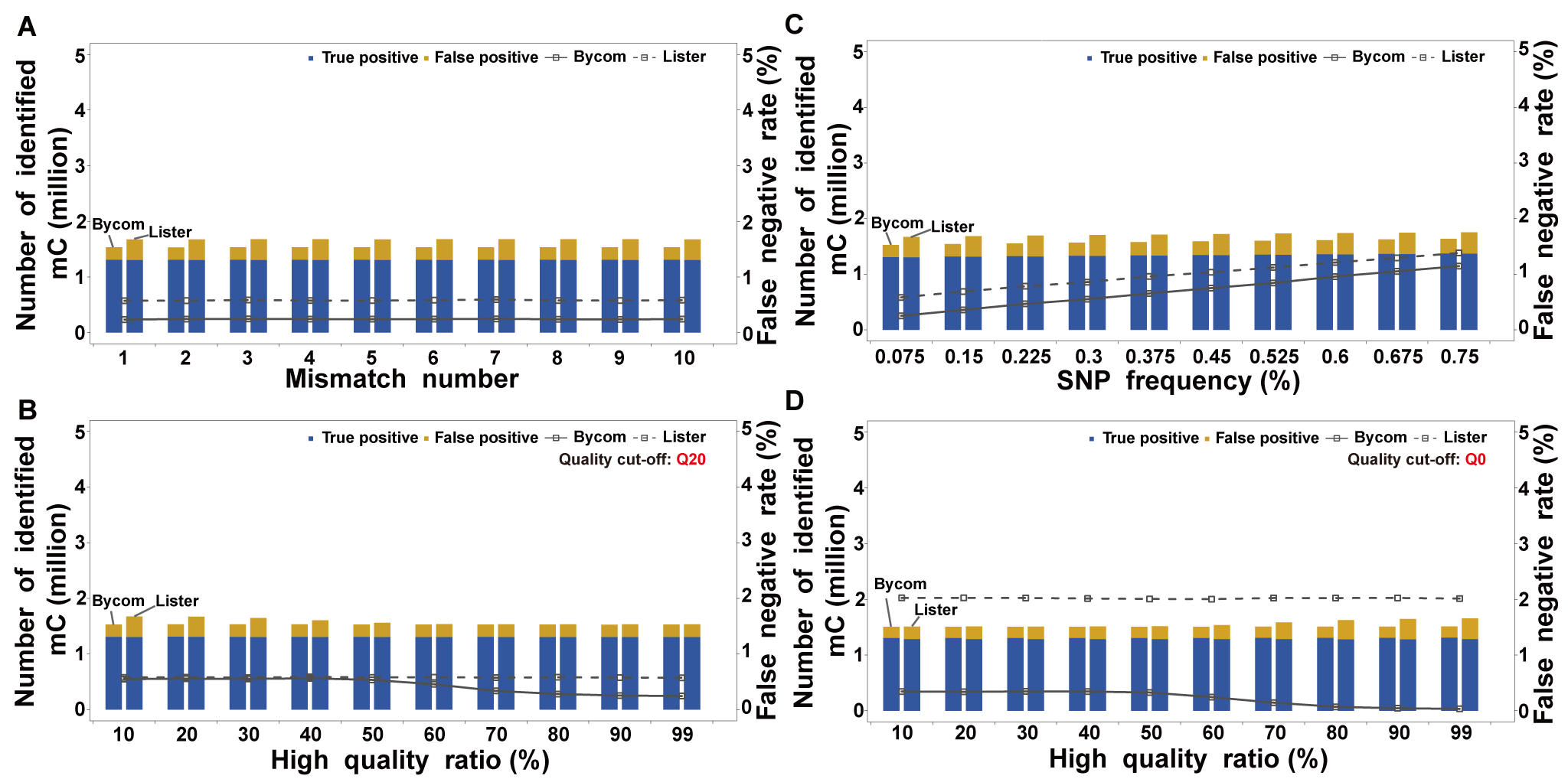

Supplement: Figure S1 — Effects of high quality value ratio (Q20), high quality value ratio (Q0), mismatch number, and SNP frequency on Bycom performance on simulated data. Performance of Bycom and Lister with a range of values for the mismatch number (A), the high quality value ratio using Q20 cut-off (B), the SNP frequency (C), and the high quality value ratio using Q0 cut-off (D). Histograms indicate the number of identified methylcytosines, and lines indicate the false negative rate. (TIF) [file pcbi.1003853.s001.tif]

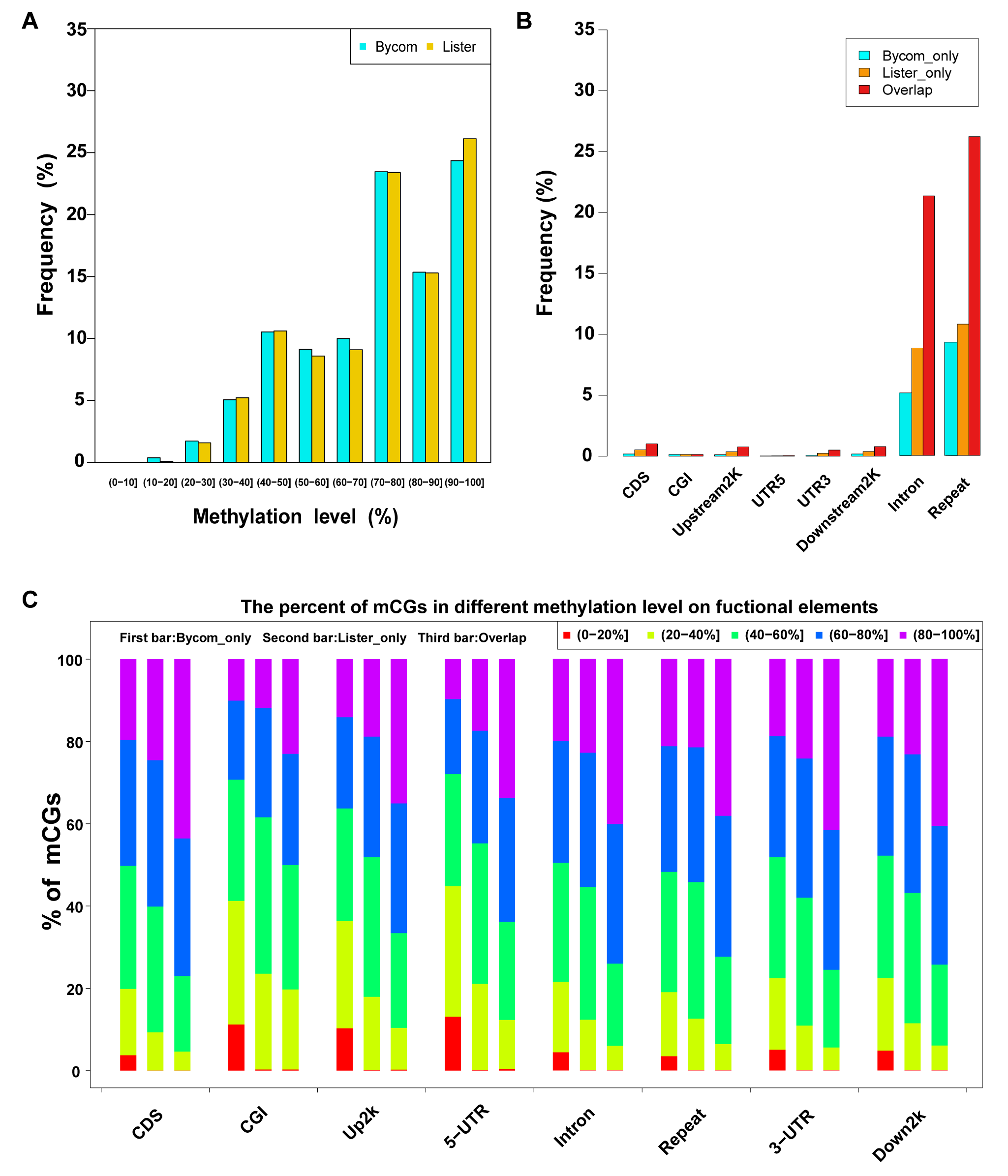

Supplement: Figure S2 — Statistic details about the methylcytosines calling on YH data. (A) Methylation level distribution of mCpGs called by Bycom and Lister. (B) Distribution of the mCpGs called by Bycom and Lister on the fuctional elements of the genome. (C) The percent of mCpGs, detected by Bycom and Lister, in different methylation level on the fuctional elements of the genome. (TIF) [file pcbi.1003853.s002.tif]
