# Supplementary material for: A Bayesian Framework to Identify Methylcytosines from High-Throughput Bisulfite Sequencing Data
Source: PLoS Comput Biol. 2014 Sep 25;10(9):e1003853. doi: 10.1371/journal.pcbi.1003853 (PMC4177668; doi:10.1371/journal.pcbi.1003853)
Supplement: Text S1 — Commands of the public software used in this manuscript. (DOCX) [file pcbi.1003853.s008.docx]

Supplementary Note 1

**Bismark**

Bismark v0.12.1 was executed with the following commands:

bismark_genome_preparation --path_to_bowtie /usr/local/bin/ --verbose fasta_dir --bowtie2

bismark --bowtie2 -N 1 -L 30 -p 8 fasta_dir fastq.file -o result_dir

bismark_methylation_extractor -s --bedGraph --counts --cytosine_report --report --comprehensive --genome_folder fasta_dir -o result_dir fastq.file_bismark_bt2.sam

**Bisulfighter**

Bisulfighter v20131226 was executed with the following commands:

python2.7 bsf-call -p 8 -o result.txt -W work_dir fasta.file fastq.file

**BSMAP**

BSMAP v2.72 was executed with the following commands:

bsmap -a fastq_1.file -b fastq_2.file -d fasta.file -o mapping_result.sam -m 100 -x 500 -S 1 -z 33 -s 16 -q 20 -f 5 -p 8 -v 0.08
